# Timely transmission of alert events and prompt management of ventricular arrhythmias using app-based remote monitoring in a patient with a cardiac implantable electronic device

**DOI:** 10.1016/j.hrcr.2026.06.039

**Published:** 2026-07-08

**Authors:** Satoshi Yanagisawa, Yasuya Inden, Michiko Hibino, Yumi Fujikake, Takahiro Okumura, Toyoaki Murohara

**Affiliations:** 1 Department of Advanced Cardiovascular Therapeutics, Nagoya University Graduate School of Medicine, Nagoya, Japan; 2 Department of Cardiology, Nagoya University Graduate School of Medicine, Nagoya, Japan; 3 Department of Clinical Engineering, Nagoya University Hospital, Nagoya, Japan; 4 Department of Cardiology, Fujita Health University School of Medicine, Toyoake, Japan; 5 Department of Cardiology, National Center for Geriatrics and Gerontology, Obu, Japan

**Keywords:** Alert event, Cardiac implantable electronic devices, Mobile app, MyCareLink Heart, Pacemaker, Remote monitoring, Smartphone, Transmission, Ventricular tachycardia

**Key Teaching Points**

• A mobile app–based remote monitoring (RM) system (MyCareLink Heart) connects patients’ devices through a downloadable app on their smartphones using low-energy Bluetooth technology, without the need for a traditional bedside console or handheld reader.
• We present a case demonstrating the clinical benefit of app-based RM for transmitting a critical ventricular arrhythmia event alert promptly, enabling quick management and effective treatment afterward.
• This case highlights the effectiveness of app-based RM in a specific situation and the evolving needs within today’s information technology society, along with the expected increase in smartphone use.

## Introduction

According to the latest guidelines, remote monitoring (RM) systems are recommended for patients with cardiac implantable electronic devices (CIEDs).^1^ RM enables early detection of clinically significant arrhythmias, heart failure (HF) exacerbations, device malfunctions, and appropriate device therapies, thereby facilitating timely intervention.^2^

Recently, mobile app–based RM technologies have emerged as potential alternatives to conventional home communicator systems.^3^, ^4^, ^5^, ^6^ The Medtronic MyCareLink Heart app (Minneapolis, MN) uses a low-energy Bluetooth-equipped electronic device and the patient’s smart device, and this system does not require a traditional bedside console or handheld reader. This system allows more timely detection of clinical events, particularly when the patient is away from home, such as during travel or business trips. Furthermore, continuous proximity between the patient and his or her smartphone facilitates more reliable transmission of scheduled data.

Herein, we present a case demonstrating the clinical utility of app-based RM in a patient with a CIED who transitioned from a conventional home communicator system. Early detection of a life-threatening arrhythmia via app-based RM enabled prompt clinical intervention and effective management.

## Case report

A 71-year-old man who had undergone cardiac resynchronization therapy with defibrillator implantation presented with multiple episodes of ventricular tachycardia (VT) transmitted via the CareLink network system (Medtronic, Inc.). 5 years earlier, he had undergone an upgrade from cardiac resynchronization therapy with pacemaker to cardiac resynchronization therapy with defibrillator because of dilated cardiomyopathy with reduced left ventricular ejection fraction and sustained VT. The patient had used a home communicator RM system (MyCareLink Relay) for 4 years and switched to an app-based RM system (MyCareLink Heart) 1 year prior, as he owned an iPhone (Apple, Inc.) (at the time, only iOS devices could connect to this app system in Japan). Despite being older than 70 years, app installation and successful connection were achieved easily without any assistance. Supplemental Figure 1 shows the CareAlert settings in this patient. The typical therapeutic zone and treatment maneuvers for VT are as previously outlined from our laboratory data.^7^

**Figure 1:**
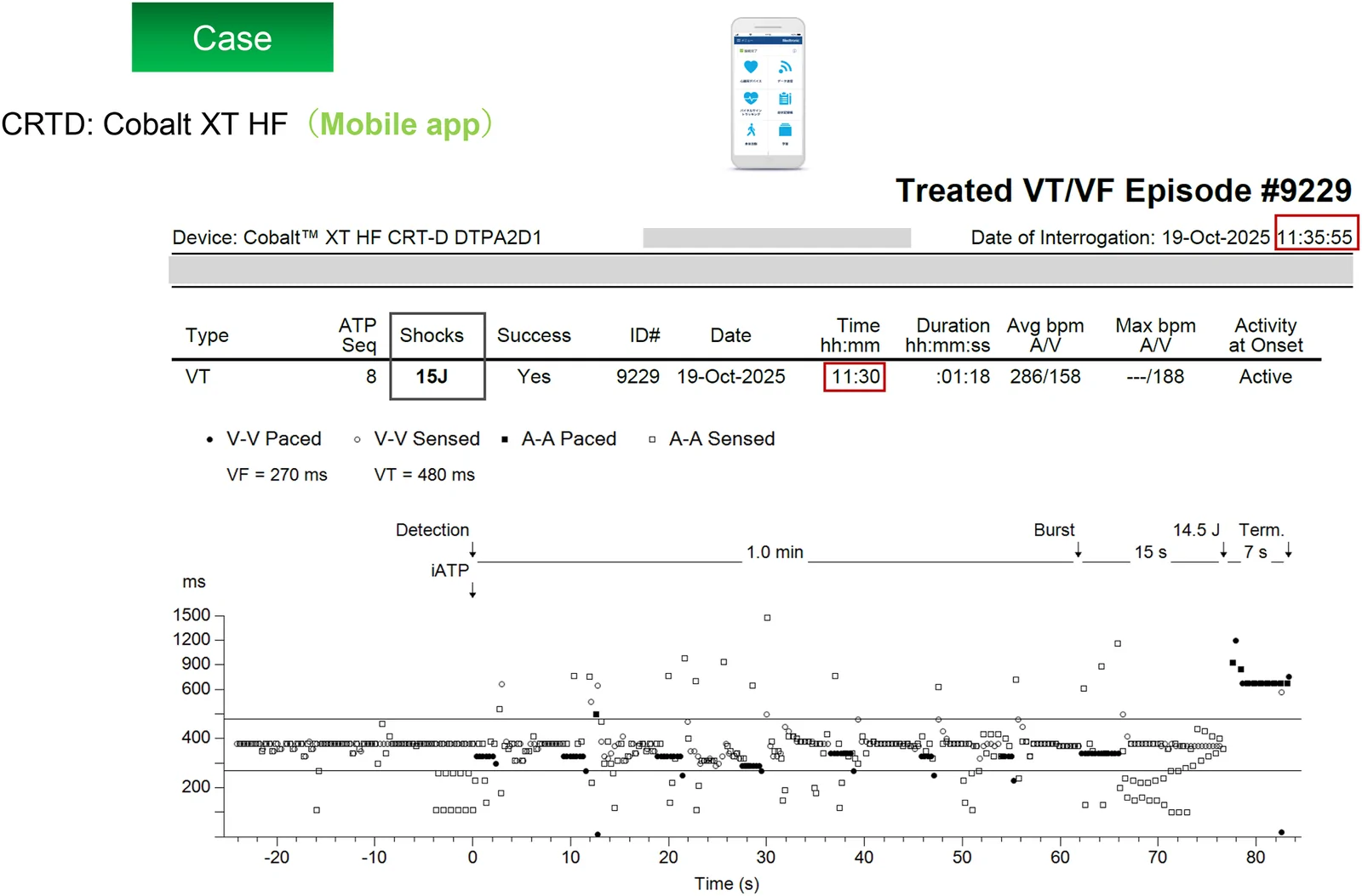
Alert event transmitted via the mobile app. A representative case of a patient with CRT-D showing an alert for shock therapy after VT refractory to multiple ATP attempts, transmitted via the mobile app. VT occurred at 11:30 AM, and shock therapy was delivered 1 minute later. The transmission occurred at 11:35 AM, with a delay of only 4 minutes from therapy delivery. Although the patient was away from home at the time of VT onset, the event was successfully transmitted via the smartphone-based mobile app. ATP = antitachycardia pacing; CRT-D = cardiac resynchronization therapy with defibrillator; HF = heart failure; iATP = intrinsic antitachycardia pacing; VF = ventricular fibrillation; VT = ventricular tachycardia.

Review of transmitted data revealed a shock therapy alert for sustained VT that was not terminated by antitachycardia pacing (ATP) (Figure 1). VT occurred at 11:30 AM, and multiple ATP therapies were delivered for ∼1 minute unsuccessfully, resulting in termination by shock therapy. The transmission time via the app was 11:35 AM, and the duration from therapy to transmission was only 4 minutes. Furthermore, at the time of VT occurrence, the patient had begun moving away from the house while carrying the smartphone with the installed mobile app; thus, the event alert was immediately transmitted to the CareLink system through the mobile app. The patient experienced persistent palpitations and the impact of the shock at the time of VT occurrence.

The event alert was reviewed ∼3 hours after transmission by the clinical engineering staff on holiday duty at our hospital, and the attending doctor contacted the patient, requesting the patient to visit the hospital as soon as possible. The patient was admitted to the hospital upon emergency arrival 1 hour later on the same day because of the frequent VT occurrences and therapeutic episodes. Echocardiography revealed severely reduced left ventricular ejection fraction (21.2%) with marked dilation. Mexiletine and sotalol were added to ongoing amiodarone therapy; however, VT recurred intermittently. Therefore, catheter ablation was performed under mechanical ventilation and intra-aortic balloon pump support. Extensive radiofrequency ablation was applied to the left ventricular outflow tract, septum, inferior wall, and lateral wall, which were suspected to be related to the isthmus of multiple VTs induced by stimulus pacing during the session. The patient was discharged without complications and remained free of VT requiring ATP or shock therapy for 4 months of follow-up.

**Figure 2:**
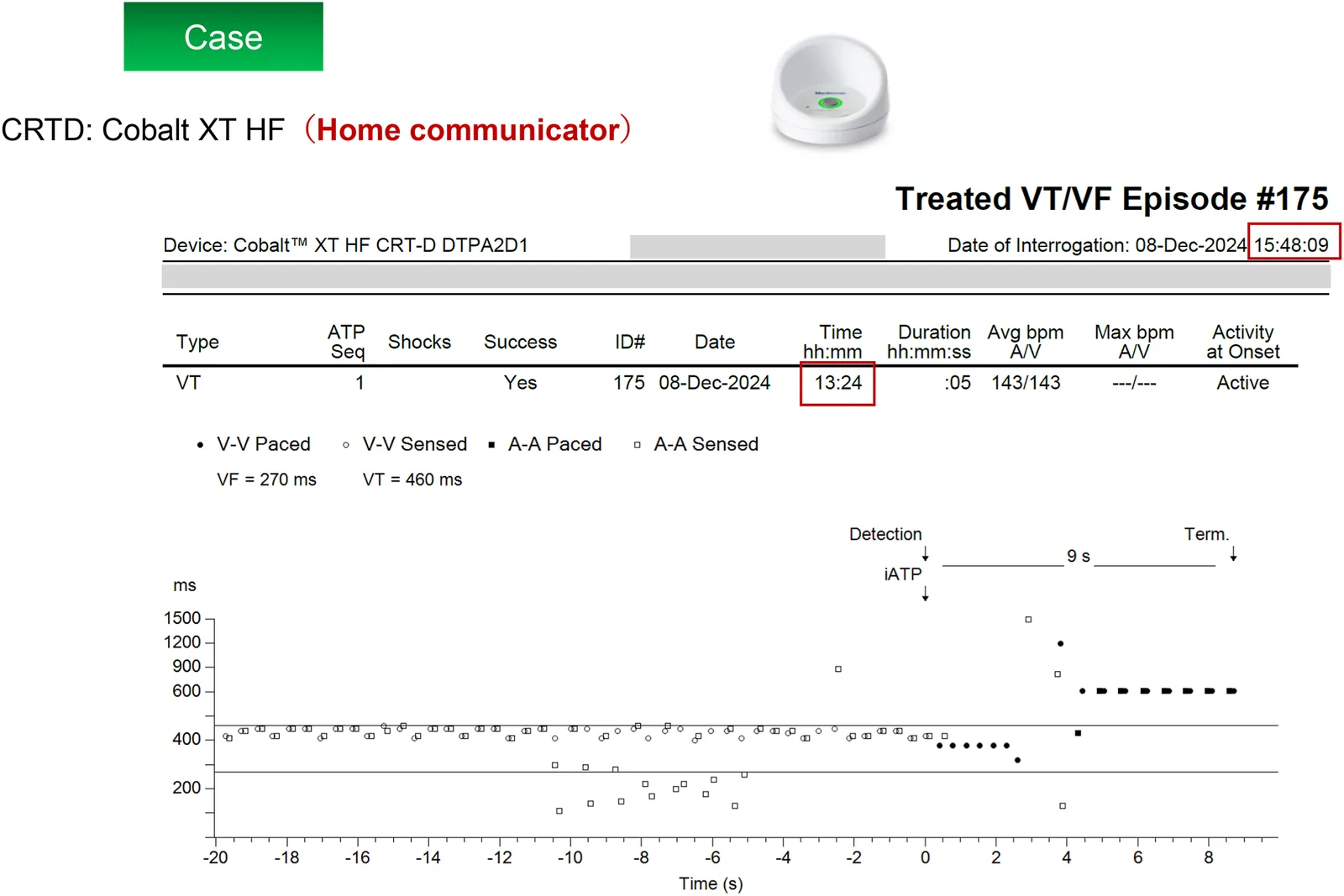
Alert event transmitted via the home communicator monitoring system. The same patient as shown in Figure 1 previously used a home communicator system before switching to the mobile app. An alert event for VT successfully treated with ATP occurred at 1:24 PM; however, transmission was delayed until 3:48 PM, resulting in a time lag of more than 2 hours. Abbreviations as in Figure 1.

**Figure 3:**
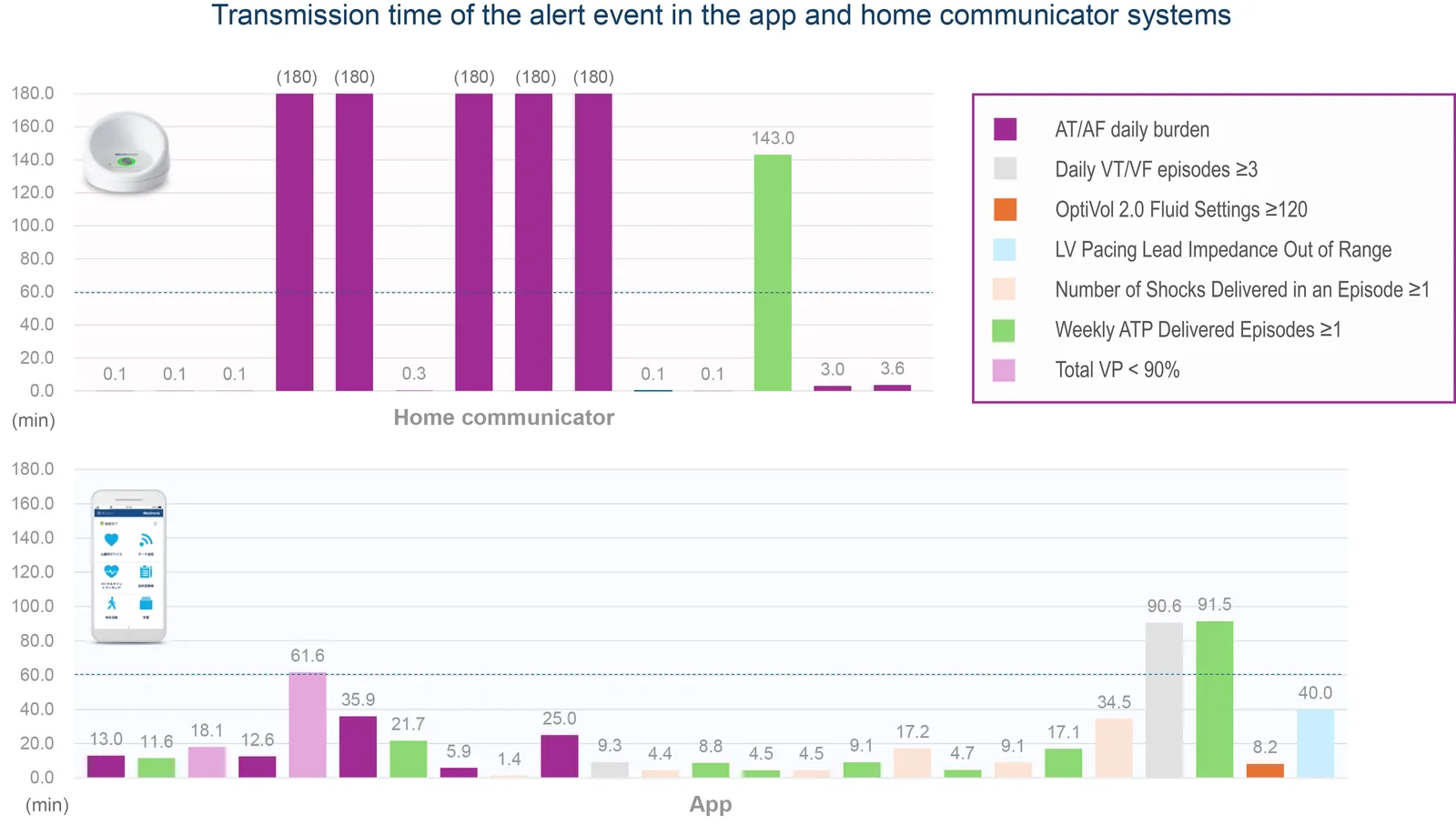
Comparison of transmission times between the app-based and home communicator systems. 25 and 14 alert events were recorded with the app-based system and home communicator system, respectively. Detailed events were 8 weekly ATP delivered alerts, 6 shock therapy alerts, 5 AF daily burden alerts, 2 daily VT/VF episodes ≥3 alerts, 2 total VP <90% alerts, 1 episode of OptiVol 2.0 (Medtronic, Minneapolis, MN) fluid settings ≥120 alert, and 1 episode of pacing lead impedance out-of-range alert. In contrast, all events were atrial fibrillation daily burden alerts except for 1 event of weekly ATP delivered alerts in the home communicator system. Because all episodes were derived from a single patient, the comparison was descriptive. The Fisher exact test was used as an exploratory analysis, and no adjustment for within-patient clustering was performed in the rate comparison. Permission to use the mobile app and home communicator images was obtained from Medtronic Japan. AF = atrial fibrillation; AT = atrial tachycardia; LV = left ventricular; VP = ventricular pacing; other abbreviations as in Figure 1.

Figure 2 represents the device log of the alert event in the same patient during the use of the home communicator monitoring system before the introduction of the app. The VT event occurred at 1:24 PM, and ATP successfully terminated the VT. However, this alert for VT was transmitted at 3:48 PM, more than 2 hours later via the home monitoring system, after successful ATP therapy for the VT. During follow-up, 25 alert events were recorded with the app-based system and 14 with the home communicator system in this patient. The median time from event occurrence to transmission was 12.6 (IQR: 7.1–29.8) minutes for app-based RM and 3.3 (0.1–180.1) minutes for home communicator RM (*P* = .831) (Figure 3). However, transmission within 60 minutes occurred significantly more frequently with the app-based system than with the home monitoring system (88% [22 of 25] vs 57% [8 of 14]; *P* = .047) (Figure 3). Among the available alert workflow data, including 25 app-based alerts and 3 home communicator alerts, the median time from transmission to staff review was 13.1 (2.9–27.9) hours for app-based alerts and 16.0 (1.7–not available) hours for home communicator alerts. Accordingly, the total time from event occurrence to staff review was 13.1 (3.1–28.3) and 16.1 (4.1–not available) hours, respectively. The patient was aligned with the scheduled monthly transmission through both RM systems during follow-up.

## Discussion

This case demonstrates the clinical utility of an app-based RM system in rapidly transmitting critical ventricular arrhythmia alerts, enabling timely intervention and effective management. The rapid transmission observed in this case was likely facilitated by the continuous proximity of the smartphone to the patient, allowing event detection within minutes, even while the patient was outside the home. This advanced system offers various perspectives to facilitate timely diagnosis, improve transmission rates, and enable early detection of events during the RM process in patients with CIEDs. It also highlights the potential for health care delivery models to evolve within today’s information technology society and the anticipated rise in smartphone use.

Currently, the Medtronic home communicator RM console (MyCareLink Relay) communicates with CIEDs using wireless technology over a wide area of <3 m, enabling high transmission success rates. Overall outcomes and utility of the bedside RM are acceptable; however, there are some unmet needs with this bedside RM in specific situations. Home-based RM systems are generally confined to the patient’s residence and do not provide connectivity outside the home. During travel or extended absence, RM is unavailable unless the patient carries the home communicator device (∼14 × 14 × 10 cm). Consequently, critical events such as sustained atrial fibrillation or ventricular arrhythmias may go undetected until the patient returns home or develops symptoms such as syncope, stroke, or HF exacerbation. Such a situation can also occur in a patient sleeping in another room, outside the bedroom, where the home communicator RM console is not located, or in patients who are hospitalized for an extended period. In contrast, app-based RM systems maintain continuous connectivity through smartphones, enabling real-time monitoring regardless of patient location. This feature is particularly advantageous for individuals who travel frequently or are often away from home. In general, installing the MyCareLink Heart app is straightforward for most smartphone users, as described in Supplemental Methods.

Our report highlighted an advantage of the app-based RM system by showing a significantly shorter alert transmission time, often just a few minutes. In addition, ∼90% of alert events were transmitted within 60 minutes after switching to the app-based RM system. These findings demonstrate that the app-based RM system can reliably transmit real-time alert events, which can increase patient’s confidence, as the RM system remains connected to the patient’s implanted device at all times. Specifically, app-based RM was effective for early transmission even after hospitalization in this case because many of the VT alert events during hospitalization were successfully sent within 60 minutes via the patient’s smartphone, which he carried during the emergency.

Early detection of ventricular arrhythmias is essential for timely intervention, prevention of HF deterioration, and avoidance of electrical storm, ultimately improving prognosis.^8^ In addition, app-based RM may offer cost advantages and reduce environmental burden by eliminating the need for dedicated hardware consoles, aligning with sustainable development goals. However, app instructions and smartphone use are sometimes not user-friendly for older patients who are unfamiliar with advanced technology devices, which could hinder the growth of this type of RM system. Although this patient independently installed the app without assistance, barriers such as limited digital literacy in older adults, smartphone access, and connectivity issues may limit broader adoption. In such cases, family members (up to 5 users) using an iOS device can connect to the patient’s RM system and monitor the patient’s status instead, which may be another benefit of the MyCareLink Heart app system.

A remaining challenge of app-based RM is the delay between alert transmission and clinical recognition/action. As highlighted in this case, although technology can reduce transmission delay, human workflow may still prolong response time, which remained comparable to the 1–2 days reported in previous studies.^2^^,^^5^ Because RM review is only 1 part of broader clinical responsibilities for nurses, clinical engineers, and physicians, real-time review is often impractical, especially during nights, weekends, or high-workload periods. Future integration of artificial intelligence–assisted alert triage, automated patient guidance, and specialized third-party RM management services may help address this operational gap. Larger prospective studies are needed to evaluate differences in response time and clinical outcomes between RM systems.

### Study limitations

This was a retrospective single-case study, limiting generalizability, with device/vendor specificity, iOS-only availability in Japan, and potential barriers related to smartphone literacy for app-based RM (MyCareLink Heart). In addition, a potential conflict of interest should be considered, as 2 authors are affiliated with a Medtronic-sponsored endowed department. The alert event characteristics differed between the app-based and home communicator systems, requiring caution in comparisons. Furthermore, the comparison shown in Figure 3 was based on repeated events from a single patient without controlled or randomized assignment; therefore, the analysis was exploratory and descriptive, without adjustment for within-patient clustering. This single-case report, with a limited number of alert events, does not allow us to demonstrate definitive improvements in clinical outcomes, total response time, or operational efficiency.

## Conclusion

This case highlights the utility of app-based RM in enabling rapid detection and transmission of ventricular arrhythmia alerts, facilitating prompt clinical management and effective treatment. App-based RM may represent a promising advancement in modern health care systems, given the continued evolution of wireless technologies and the widespread adoption of smartphones. However, rapid transmission alone does not guarantee a rapid clinical response unless paired with reliable alert review workflows, requiring a well-organized workflow system and staff cooperation.

## Disclosures

Drs Yanagisawa and Okumura are affiliated with a department sponsored by Medtronic Japan. The rest of the authors have no conflicts of interest.
