# Supplementary material for: Timely transmission of alert events and prompt management of ventricular arrhythmias using app-based remote monitoring in a patient with a cardiac implantable electronic device
Source: HeartRhythm Case Rep. 2026 Jul 8;12(9):1063–7. doi: 10.1016/j.hrcr.2026.06.039 (PMC13593530; doi:10.1016/j.hrcr.2026.06.039)
Supplement: Supplementary file 1 [file mmc1.docx]

**Supplemental Materials**

Yanagisawa *et al.*: Timely transmission of alert events and prompt management of ventricular arrhythmias using app-based remote monitoring in a patient with cardiac implantable electronic devices

**Table of contents**

***Page 2: Supplemental Methods.*** Introduction and demonstration of the MyCareLink™ Heart app

***Page 3: Supplemental Figure 1.*** Alert settings for device tone and wireless notifications

**Supplementary Methods**

***Introduction and demonstration of the MyCareLink™ Heart app***

Detailed instructions for app installation follow the standardized leaflet provided by Medtronic.

Patients first accessed the MCLHeart.com website by scanning a QR code with their personal smartphones. When the “Get” button appeared on the screen, they tapped it to download the application. Once the download was complete, the MyCareLink™ Heart app icon appeared on the device screen.

During the setup process, patients entered their first and last names, email address, and a password for registration. They then input the serial number of their implanted cardiac device. After device pairing, patients opened a confirmation email sent to their registered email address. By selecting the verification link in the email, they were directed to a webpage where they re-entered their email address and password to complete the verification process.

After successful registration, patients returned to the home screen and launched the application. The MyCareLink™ Heart app is compatible with the following Medtronic devices: Cobalt™ XT ICD, Cobalt™ MRI CRT-D, Azure™ MRI, Percepta™ MRI CRT-P, and LINQ II™. Currently, in Japan, only iPhone devices (Apple Inc.) are supported by this app system.

***Supplementary Figure 1. Alert settings for device tone and wireless notifications***

Alert settings configured for the CRT-D device in the present patient.
AF, atrial fibrillation; AT, atrial tachycardia; ATP, anti-tachycardia pacing; CRT-D, cardiac resynchronization therapy defibrillator; LV, left ventricle; RV, right ventricle; SVC, superior vena cava; VF, ventricular fibrillation; VT, ventricular tachycardia.
